# Protease-Specific Biomarkers to Analyse Protease Inhibitors for Emphysema Associated with Alpha 1-Antitrypsin Deficiency. An Overview of Current Approaches

**DOI:** 10.3390/ijms22031065

**Published:** 2021-01-21

**Authors:** Simona Viglio, Elisabeth G. Bak, Iris G. M. Schouten, Paolo Iadarola, Jan Stolk

**Affiliations:** 1 Department of Molecular Medicine, University of Pavia, Via Taramelli 3, 27100 Pavia, Italy; 2 Department of Pulmonology, Leiden University Medical Center, Albinusdreef 2, 2333 Leiden, The Netherlands; e.g.bak@umail.leidenuniv.nl (E.G.B.); i.g.m.schouten@lumc.nl (I.G.M.S.); J.Stolk@lumc.nl (J.S.); 3 Department of Biology and Biotechnologies “L. Spallanzani”, University of Pavia, Via A. Ferrata 9, 27100 Pavia, Italy; piadarol@unipv.it

**Keywords:** alpha1-antitrypsin, AAT, alpha1-antitrypsin deficiency, AATD, human neutrophil elastase, proteinase 3, AAT replacement therapy, synthetic NE inhibitors, synthetic proteinase 3 inhibitors

## Abstract

As a known genetic cause of chronic obstructive pulmonary disease (COPD), alpha1-antitrypsin deficiency (AATD) can cause severe respiratory problems at a relatively young age. These problems are caused by decreased or absent levels of alpha1-antitrypsin (AAT), an antiprotease which is primarily functional in the respiratory system. If the levels of AAT fall below the protective threshold of 11 µM, the neutrophil-derived serine proteases neutrophil elastase (NE) and proteinase 3 (PR3), which are targets of AAT, are not sufficiently inhibited, resulting in excessive degradation of the lung parenchyma, increased inflammation, and increased susceptibility to infections. Because other therapies are still in the early phases of development, the only therapy currently available for AATD is AAT augmentation therapy. The controversy surrounding AAT augmentation therapy concerns its efficiency, as protection of lung function decline is not demonstrated, despite the treatment’s proven significant effect on lung density change in the long term. In this review article, novel biomarkers of NE and PR3 activity and their use to assess the efficacy of AAT augmentation therapy are discussed. Furthermore, a series of seven synthetic NE and PR3 inhibitors that can be used to evaluate the specificity of the novel biomarkers, and with potential as new drugs, are discussed.

## 1. Introduction

Despite thousands of people in the world being diagnosed with the autosomal co-dominant disease alpha1-antitrypsin deficiency (AATD), this condition is estimated to be highly underdiagnosed; the low awareness of physicians is perceived as the greatest barrier to the diagnosis rate [1]. AATD is characterised by intra- and extra-pulmonary diseases. The intra-pulmonary diseases include lung emphysema, bronchiectasis, and chronic bronchitis. Due to the resulting fixed airflow obstruction, most individuals with AATD experience symptoms of dyspnoea, exercise intolerance, and fatigue. Furthermore, these patients are more prone to infections, and inflammation is normally present in their lung tissue. The extrapulmonary diseases include liver dysfunction, panniculitis, granulomatosis with polyangiitis (GPA), and ulcerative colitis, the latter of which occur only rarely. Based on the irreversible airflow obstruction, limited gas exchanges, symptoms, and medical history, patients are diagnosed with chronic obstructive pulmonary disease (COPD), a disorder that typically occurs in smokers. However, while COPD usually develops at an advanced age in smokers without AATD, it mostly occurs around the age of 30–50 years old in AATD patients [2,3,4]. Even though smoking has been indicated as the primary trigger for the early development of COPD in individuals with AATD, the disorder can also occur at a younger age in AATD patients who never smoked compared to healthy individuals [5,6]. The mutant protein central to AATD is alpha 1-antitrypsin (AAT), an antiprotease which is primarily functional in the lung [3]. To obtain a comprehensive overview of the disease, the current literature on the pathophysiology of AATD, including AAT and its targets, are summarised. Furthermore, AAT augmentation therapy is discussed, and its controversies considering its effectiveness are addressed. In addition, with the aim of outlining ways to create new strategies for the development of novel assays for measuring the effectiveness of AAT therapies, biomarkers of AAT activity and different protease inhibitors are reviewed.

## 2. Alpha 1-Antitrypsin and Its Targets—Neutrophil Elastase and Proteinase 3

**Figure 1:**
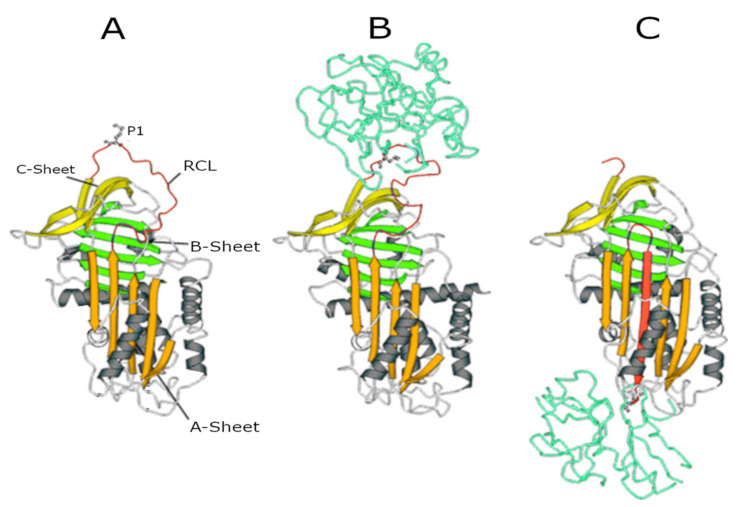
Serpin structures. (**A**) Native alpha1-antitrypsin (AAT); (**B**) Michaelis complex between AAT and trypsin; and (**C**) covalent complex between AAT and trypsin. In all structures, the A-sheet is in orange, the B-sheet is in green, and the C-sheet is in yellow. The reactive centre loop (RCL) in the upper pole of the molecule shows the P1 residue (Met358) recognised by NE.

While the majority of AAT is synthesised in hepatocytes, it is also synthesised (at much lower amounts) in neutrophils, macrophages, intestinal and pulmonary alveolar, airway epithelial cells, and in the cornea [7,8]. The gene encoding AAT is *SERPINA1* (*14q32.1*), which consists of seven exons and six introns. After translation, transcription, and post-translational modifications, AAT is released into the circulation. Via the circulation, it reaches the lungs where it functions as a serine protease inhibitor [9]. In this function, it controls the amount of proteolytic degradation, primarily by targeting the neutrophil-derived serine proteases neutrophil elastase (NE), proteinase 3 (PR3), and cathepsin G (Cath G) [10,11]. The central domain of AAT in the inhibitory process is the reactive centre loop (RCL), an amino acid sequence which is crucial for protease recognition and binding. The AAT-protease docking results in a Michaelis complex, the ‘stressed’ AAT conformation, allowing for proteolysis of the RCL. Cleavage of the RCL flips the protease to the opposite pole of AAT, making it kinetically trapped. Simultaneously, AAT ‘relaxes’ again by entering a stable state (see panels A to C in Figure 1), which can be removed from the circulation.

The fact that AAT is cleaved during this process makes it a single-use inhibition mechanism [7,12,13]. A crucial amino acid in this process is the methionine at position 358 of the enzyme because it is involved in the cleavage of the RCL and the flipping of the protease. This methionine residue, however, is also susceptible to oxidation to methionine sulphoxide. Oxidation, which can result from exposure to endogenously produced reactive oxidants derived from inflammatory cells or exposure to oxidants present in inhaled toxicants such as cigarette smoke, reduces the affinity of AAT for proteases and inactivates AAT [7].

Neutrophil elastase and proteinase 3 are the proteases which are inhibited by AAT and are most important in the development of the AATD phenotype. Cathepsin G, the third proteinase that is cleaved by AAT, does not seem to be involved in this process and will therefore not be considered in this report further [10,11]. Both NE and PR3 are synthesised in neutrophils and stored in an active form in azurophilic granules of the neutrophil [14]. Upon stimulation, neutrophils degranulate and expose the environment to the instantly active proteinases. Upon degranulation, these proteases can be present in a membrane-bound or a soluble form [14,15]. Once released, NE and PR3 are free to cleave their substrates. As these enzymes have 54% of their sequence in common, their substrate specificity is quite similar; both prefer cleaving peptide bonds after small hydrophobic amino acids [16]. Nonetheless, there still are variations in enzyme–substrate interactions, mainly due to differences in the structure of the part of the peptide surrounding the cleavage point (extending beyond P1′; Schechter and Berger nomenclature [17]) [18].

Although almost all extracellular matrix proteins can be cleaved by NE, clotting factors, complement proteins, immunoglobins, and cytokines are also amongst the targets of this enzyme [19]. When expressed at normal levels, NE predominantly has protective effects in host defence against infection. It aids in the degradation of pathogens by blocking the growth of Gram-negative bacteria [20], fine-tunes tissue remodelling [21], and is involved in the formation of neutrophil extracellular traps [22]. Although NE has beneficial effects when expressed in normal levels, overstimulation of NE production and excessive release cause excessive degradation of extracellular matrix proteins like elastin, collagen, and fibronectin, and cell-associated proteins like E-cadherin. This results in lung parenchyma degradation and disruption of the epithelial barrier due to loss of integrity and shedding of epithelial cells. These processes, when uninhibited, will result in emphysema and chronic inflammation [14,23].

Whereas plenty of literature is available concerning the function of NE, less information is available on the role of PR3. Because PR3 has many of the same targets as NE, it also proteolytically cleaves extracellular matrix proteins. Apart from that, PR3 is involved in early apoptosis [16]. Interestingly, the majority of the literature on PR3 outlines its involvement in GPA, one of the extrapulmonary diseases associated with AATD. GPA is characterised by a high expression of membrane-bound PR3 on neutrophils, which is recognised as an antigen by anti-neutrophil cytoplasmic antibodies (ANCAs) typically present in such patients. In patients with AATD, the lack of AAT causes an increase of active PR3 at the cell surface of neutrophils and thereby increases the risk of developing GPA [24]. This is one example of the important role that AAT has in regulating the balance of proteolytic activity in the lungs. Inhibiting NE and PR3 and thereby preventing unintended inflammation and damage to the lung tissue is a crucial function of AAT, and important for maintaining lung health. Immediately after degranulation, however, NE and PR3 outnumber AAT in the zone close to the cell membrane. In addition, AAT is not able to entirely reach the membrane of the neutrophils, so membrane-bound NE and PR3 will not be inhibited as effectively [25]. This indicates that the control of proteolysis by NE and PR3 is the result of a delicate balance between these proteases and AAT.

An antiprotease which can possibly take on the function of AAT when AAT is absent is alpha2-macroglobulin (α2-M) [11]. As demonstrated in earlier studies, α2-M is capable of irreversible NE inhibition [26]. Although sequestering of NE by α2-M is not very common in healthy individuals because AAT is more effective, it does seem to be important in NE control when AAT is absent [27]. Inhibition of PR3 by α2-M, on the contrary, is poor [28].

## 3. Alpha-1-Antitrypsin Properties beyond the Protease-Antiprotease Balance Hypothesis

Studies have shown that purified AAT may modulate immune cell function of neutrophils [29], monocytes [30], and T cells [31]; cells that are all relevant in the pathogenesis of pulmonary emphysema. Physiological concentrations of AAT are probably required to protect against overshooting of inflammation. In the past ten years, several mechanisms have been identified, with each capable of mediating cytokine-driven chemotaxis of inflammatory cells. Bergin et al. identified that AAT inhibits the chemotactic response of both CXCR1 and FcγRIIIb receptor signalling involved in IL-8 activity [32]. Interleukin-1β production by inflammatory cells requires signalling by both LPS and extracellular ATP. Grau et al. showed that physiological concentrations of AAT inhibit the ATP-induced release of IL-1β by human monocytic cells [33].

Lockett et al. reported that AAT modulates the inflammatory responses of lung endothelial cells to TNF-α in a complex manner [34]. AAT downregulates TNF-α gene expression by inhibiting the nuclear factor kappa-light-chain-enhancer of activated B cells (NF-κB) signalling.

Other anti-inflammatory effects of AAT were presented in a review by Janciauskiene et al. [35].

## 4. The Pathophysiology of Alpha 1-Antitrypsin Deficiency

In the case of AATD, a mutation in *SERPINA1* causes the production of a dysfunctional AAT or absence of AAT, resulting in a lower circulating amount of AAT. As mentioned before, this can severely damage the lungs as a consequence of the unlimited proteolysis of substrates by NE and PR3 [36]. More than 125 single-nucleotide polymorphisms (SNPs) are known for the AAT allele, the most well-known being the normal allele M and the Z-, S- and null-mutations [36,37]. The Z-mutation (Glu342Lys) encodes a misfolded AAT (Z-AAT), which accumulates in the endoplasmic reticulum (ER) of the hepatocytes and spontaneously polymerises intra- and extracellularly [10,38].

By triggering the release of pro-inflammatory cytokines in response to ER stress and overload resulting from the accumulation of the misfolded Z-AAT in the ER, and by the pro-inflammatory activity of the released polymers, the Z-mutation contributes to inflammation and development of liver disease [9]. Moreover, since these processes result in a marked decrease in circulating AAT, lung disease develops [10]. The S-mutation (Glu264Val) also causes the formation of polymers but at a slower rate. These polymers are less harmful because they retain fewer AAT molecules and are often degraded intracellularly already. The serum levels of AAT in patients with the S-mutation are therefore usually above the protective threshold [39]. The null-mutations, often being nonsense or frameshift mutations due to gene deletions and intron mutations, result in a complete absence of AAT in the circulation [9]. This often results in a more severe form of AATD in which COPD can already occur under the age of 30. These underlie the respiratory problems but leave the liver unaffected [36,37]. Due to the involved mutations, AATD is the only proven genetic risk factor for the development of COPD [3].

Inflammation in AATD is the result of the lack of the anti-inflammatory and immunomodulatory effects of AAT, which it carries out besides its anti-protease effects. One of the main cytokines involved in this inflammation is TNF-α. In the presence of reduced levels of AAT, TNF-α will recruit neutrophils, promote degranulation of these neutrophils, and increase apoptosis in endothelial cells and neutrophils [40]. As a consequence of the enhanced degranulation, the NE and PR3 levels will rise, causing the patients with AATD to end up with degradation of the lung matrix and emphysema [3,41]. If AAT is present, however, it inhibits the release of TNF-α via modulation of TNF-α receptor1 and TNF-α receptor2 signalling, amongst other pro-inflammatory cytokines, and stimulates the release of anti-inflammatory cytokines by innate immune cells. In addition to this, AAT can control the levels of reactive oxygen species (ROSs) by acting as a ROSs scavenger. If ROSs levels are increased, more damage to the lung parenchyma will occur and inflammation will increase [3,42]. As a result of these anti-inflammatory and immunomodulatory signals, AAT reduces the influx of neutrophils and thereby inflammation.

## 5. AAT Augmentation Therapy and Its Alternatives

AAT augmentation therapy involves the supplementation of plasma-purified human AAT to patients affected by AATD to reach the protective serum threshold of 11 µM. The two-year rapid randomized controlled trial (RAPID-RCT) study used a series of outcomes to study the effects of this therapy, i.e., the annual rate of decrease in lung density, as assessed by CT as the primary outcome, and forced expiratory volume in one second (FEV1) and AAT concentrations as secondary outcomes. A slight but controversial significant change in lung density in the long-term has been demonstrated notwithstanding an improvement in lung function has not been detected. On the other hand, the AAT levels were significantly increased, meaning that the supplementation did not result in AAT degradation [36]. In a follow-up study, the rapid open-label extension (RAPID-OLE) trial surveyed the participants of the RAPID-RCT study for another two years, confirming its results. Furthermore, a significant inflection point in lung density loss for patients who started the AAT supplementation at the start of RAPID-OLE has been observed, indicating that the disease progression was delayed by AAT supplementation [43]. In addition, in a meta-analysis that had the difference in FEV1 rate of decline as the primary outcome, the rate of loss of lung function in a subgroup of patients with AATD was decreased by AAT supplementation [44]. Even though the efficiency of AAT augmentation therapy has been assessed by some investigators, others argue that this cannot be claimed [45,46] due to controversial results obtained, and due to the fact that its effect on the protease-antiprotease balance is still not fully understood. Consequently, the AAT augmentation therapy has only been implemented in a few countries.

Various studies using other therapies are being carried out. The so-called inhaled AAT therapy involves the inhalation of human plasma-derived AAT, which is promptly deposited into the lungs. Despite its efficacy, clinical studies are still needed to determine optimal inhalation devices and AAT doses [47]. Gene therapy has been investigated by using different methods of altering gene expression. These promising approaches involve i) the transfection of the M-AAT gene by using a viral vector [48], ii) the use of a transposon to correct the point mutations in *SERPINA1* [49], and iii) the use of siRNA to interfere with the Z-AAT mRNA leading to its degradation [50]. The studies concerning these innovative therapies are still in an early phase and different clinical trials are underway to establish the best administration route.

### 5.1. Biomarkers of Neutrophil Elastase and Proteinase 3 Activity

The uncertainties about the efficacy of the AAT augmentation therapy urges researchers to look for other ways to assess whether this treatment should be more widely used. Instead of measuring lung density or function, in vivo determination of AAT activity would provide more convincing evidence for the effects of the therapy. AAT activity can be evaluated by measuring whether and at which rate NE and PR3 are inhibited. However, measuring the in vivo activity of NE and PR3 is difficult because they both bind to inhibitors or substrates rapidly after being released. In this respect, biomarkers of NE and PR3 activity resulting in a specific detectable cleavage product are more useful for assessing the AAT activity.

Biomarkers of NE activity include cleavage products of E-cadherin, elastin, and fibrinogen, all substrates of NE [23]. The cell-associated E-cadherin is located on epithelial cells, and NE substantially contributes to its degradation in injured lungs. This cleavage was marked by a specific 80 kDa fragment which could be measured in the bronchoalveolar lavage (BAL). However, PR3 and Cat G were also capable of generating this fragment, and therefore the specificity of this biomarker is not considered to be very high [23].

More promising footprints of NE activity were revealed after studying the degradation of elastin by NE. In a study performed by Kristensen et al. [51], an assay was developed for measuring the levels of an elastin fragment (EL–NE) that was cleaved between Val334 and Gly335. This fragment was specific for NE, and the levels by which it was generated by matrix metalloproteinase (MMP)-2, -7, -9, and -12, and Cat G were detectable but neglectable. The recovery of this fragment from human serum upon ex-vivo addition to serum was between 85% and 104%, and the levels of EL–NE from patients affected by idiopathic pulmonary fibrosis and lung cancer correlated with their disease. A subsequent study concluded that the EL–NE levels were associated with the FEV1 (expressed as % predicted) of COPD patients. Also, the EL–NE levels were associated with the self-rating Medical Research Council (MRC) scale of disability due to breathlessness [52] and increased in both moderate and severe exacerbations [53]. Even though no data were available on whether PR3 could also generate this neoepitope, the assay seems to be a valid method of evaluating the NE and thus the AAT activity.

The elastin degradation product ELP-3 can also be used as a measure of PR3 activity. A special assay for this neoepitope has been developed and validated. Additionally, tests of NE showed that NE only generated the same fragment at low concentrations. Moreover, COPD patients displayed higher ELP-3 concentrations than healthy individuals, making this fragment a promising footprint as well [54]. Other markers of elastin degradation are desmosine and its isomer isodesmosine. Levels of these amino acids can be quantified by radioimmunoassay or by HPLC analysis of the urine, as both amino acids are filtered by the kidney [55,56].

Finally, fibrinogen is also cleaved by NE, resulting in multiple NE-specific neoepitopes. Fibrinogen is a glycoprotein with a total molecular mass of about 340,000 Da. It consists of three pairs of chains, the Aα-, the Bβ- and the γ-chains, which are twisted around each other [57]. A NE-specific neoepitope that is generated from the Bβ-chain is Bβ 30–43. This fragment has been used in different studies to measure the activity of NE but will most likely not be of use in determining NE activity in patients with AATD because AAT is necessary to generate this peptide. In the absence of AAT, another peptide with high affinity for the Bβ 30–43 antibody will be produced, disturbing the measurements for the Bβ 30–43 antibody [58,59]. The Aα-chain has been found to be the source of two other neoepitopes that can be measured in human blood. The first one is Aα 1–21, which appears specific to NE, although its generation by PR3 has not been investigated. Interestingly, NE could still be inhibited by AAT when it was already interacting with fibrinogen. Thus, an effect of AAT administration is likely to be immediately detected. A disadvantage of the assay for this fragment is that it requires the administration of thrombin to increase the immunoreactivity [60]. Furthermore, in vivo, the Aα 1–21 fragment easily degrades to Aα 1–20 or Aα 1–19 [61]. A more stable fragment generated by the cleavage of the Aα-chain of fibrinogen is Aα-Val360. This footprint can exclusively be produced by NE, and even PR3 is known to be unable to generate it. It therefore gives a reliable reflection of the activity of NE. In addition, Aα-Val360 is formed at the site of release of NE, making it a pre-inhibition neoepitope. A PR3-specific fibrinogen epitope that is as promising and similar to Aα-Val360 is Aα-Val541. This fragment is also rather stable, can be measured in the blood of individuals, and is generated at the point of release of PR3 [62].

### 5.2. Synthetic Small Molecular Weight Inhibitors of Neutrophil Elastase and Proteinase 3

As shown in Table 1, several inhibitors have been developed over the last years, and each inhibitor has different properties concerning binding sites, cell-permeability, and potency.

Seven synthetic small molecular weight serine protease inhibitors are discussed on these properties according to the generations they evolved in [25]. Because it is often the case that only the interactions between the inhibitor and NE are known, and not the interactions between the inhibitor and PR3, only the interaction with NE is described. The first generation of inhibitors includes the natural inhibitors of NE and PR3, AAT, and A2M, which have already been discussed in this review article.

**Figure 2:**
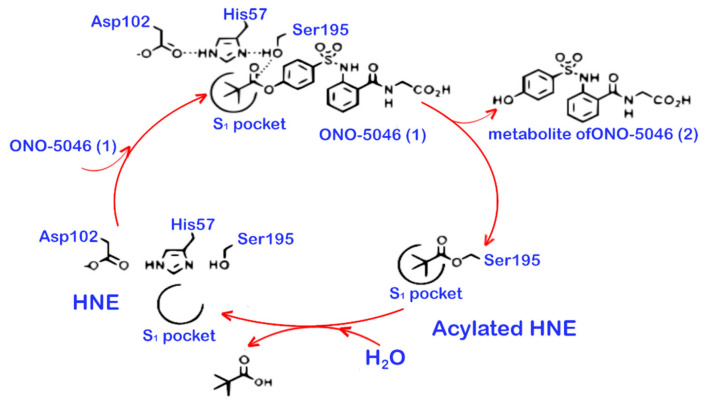
The proposed mechanism of NE inhibition by ONO-5046.

The second generation of inhibitors includes the first synthesised small molecule inhibitors with active-site recognition. Small molecule inhibitors are the preferred type of inhibitors because they are able to reach the cell surface to inhibit membrane-bound proteases and/or to cross the cell membrane. Natural inhibitors are not able to achieve this. Most inhibitors of the second generation act based on mechanism, i.e., by covalently attacking the active site of the NE or PR3. Two inhibitors of this generation have been developed by ONO Pharmaceutical (Chuo-ku, Osaka, Japan). The first one is ONO-5046, also known as Sivelestat. Its interaction with NE is mediated by hydrogen bonds and hydrophobic interactions. These interactions take place in subsite 1 (S1) of the active site of NE. The main hydrogen bonds are established between ONO-5046, Ser195, and Gly193 [63]. During the reaction with NE, ONO-5046 is transformed, and a part of the inhibitor remains in the S1 pocket and another part is released as a metabolite. The part which is left in the S1 pocket can be deacylated and removed from the pocket again (see Figure 2).

The interaction between ONO-5046 and NE thus is reversible, and ONO-5046 can only be used once [64]. Furthermore, ONO-5046 cannot effectively pass the cell membrane [65]. The Ki of ONO-5046 inhibiting NE is 200 ± 20 nM. Experiments with NE from rabbits, rats, hamsters, and mice showed that ONO-5046 can also successfully inhibit NE from these species. At the same time, ONO-5046 is inactive against bovine pancreas trypsin, human plasma thrombin, human plasma plasmin, porcine and human pancreas kallikrein, bovine pancreas chymotrypsin, and human neutrophil cathepsin G [66]. On the contrary, inhibition of PR3 was successful [67]. After several clinical trials, ONO-5046 was approved as a treatment for patients with acute lung injury and systemic inflammatory response syndrome in Japan and Korea [68]. This approval is based on results that indicate that ONO-5046 improves lung function in these patients [69,70]. Other studies, however, showed contradictory results and none of the studies could demonstrate a change in 30-days survival [71,72]. Therefore, other countries have not approved ONO-5046 as a drug.

Another drug in this generation is ONO-6818, also known as Freselestat. Just like ONO-5046, ONO-6818 also covalently attacks Ser195 and uses hydrogen bonds to stabilise the connection to NE. The established bond between the two molecules is reversible. ONO-6818 is specific for NE, and it is inactive against PR3, trypsin, pancreatic elastase, plasmin, thrombin, type I collagenase, cathepsin G, and murine macrophage elastase. The Ki-value of NE inhibition by ONO-6818 is 12.16 nM [73]. Although ONO-6818 is not cell-permeable, it is capable of reducing IL-8 levels. This might prevent degranulation of the neutrophils and therefore administration of this inhibitor can result in the reduction of NE levels [74].

The third and fourth generations of NE and PR3 inhibitors closely resemble each other. The third-generation inhibitors bind the S1 pocket of NE and thereby trigger the deepening of subsite 2 (S2) of the active site. This allows for further interactions with the inhibitor, which occur with inhibitors of the fourth generation. By interacting with the S2 pocket, these inhibitors are able to more specifically target NE. Two potent inhibitors of these generations are AZD9668 and BAY-678. AZD9668 reversibly inhibits NE with a Ki of 9.4 nM. It was found to be ineffective against PR3 and Cath G, as well as to bovine chymotrypsin, porcine pancreatic elastase, and bovine trypsin. Its inhibition of NE in rats, mice, guinea pigs, and dogs was successful. Due to the fact that AZD9668 has an association constant similar to AAT, the inhibitor is likely to be an effective substitute of AAT in the case of AATD. Phase II clinical trials did not however indicate beneficial effects of AZD9668 in COPD patients on computed tomography (CT) measures [75], FEV1 [76], and respiratory signs and symptoms [77]. In contrast, a phase II clinical trial in patients with bronchiectasis did demonstrate a significant increase in lung function and a non-significant trend in respiratory symptoms favouring AZD9668 [78].

BAY-678, the other commonly used NE inhibitor of these generations, also uses reversible induced-fit binding to the active site of the enzyme [79]. It has a Ki-value of 9.4 nM and low potency against rat neutrophil elastase [80]. In addition to this, BAY-678 showed no inhibition of Cath G, porcine pancreatic elastase, chymotrypsin, and 18 other NE-related enzymes. The inhibitory activity against PR3, however, had not been tested [80].

The fifth generation of NE and PR3 inhibitors is closely related to the fourth generation. This generation of inhibitors carries an additional substituent which freezes the structure in the ideal conformation for binding the protease. The freezing consists of blocking the ability of the inhibitor to rotate around the pyrimidinone-cyanophenyl axis. The locked confirmation caused by the freezing thus anticipates the inhibitor for binding to the enzyme [25]. An example of such an inhibitor is BAY 85-8501. Because the previously discussed BAY-678 and BAY 85-8501 were examined in the same study [80], BAY 85-8501 was tested for activity against the same enzymes to determine its selectivity, and it also proved to be inactive against the same 21 enzymes. Additionally, its inhibitory activity against PR3 is unknown. Nonetheless, BAY 85-8501 turned out to be very potent with a Ki of 0.08 nM. Its residence time was about 17 minutes, which is considerably less than AAT, but BAY 85-8501 was able to bind NE as rapidly as AAT. In vivo, BAY 85-8501 demonstrated to be effective against the development of lung damage in mice. However, in clinical phase IIA trials, BAY 85-8501 failed to achieve clinical benefits in patients with non-cystic fibrosis bronchiectasis [81].

An inhibitor of which the details of its interaction with NE still need to be investigated is DMP-777, also known as L-694458. It is a β-lactam elastase inhibitor which is able to pass the cell membrane. Hence, this inhibitor is able to inhibit NE intra- and extracellularly [82]. Merck and Co. (Rahway, NJ, USA) and DuPont Merck Pharmaceuticals co-developed the dual NE & PR3 low molecular weight inhibitor DMP-777. DMP-777 is a cell penetrant inhibitor that inactivates both NE and PR3 within the azurophilic granules of neutrophils. DMP-777 is a potent, essentially irreversible mechanism-based inhibitor of NE, possessing a Kinact/Ki = 3,800,000 M^−1^ s^−1^ [83]. DMP-777 binds to PR3 much more slowly, possessing a Kinact/Ki = 77,000 M^−1^ s^−1^. (United States Patent No. 5,952,321, 14 September 1999.). While DMP-777 binds significantly less rapidly to PR3, both NE and PR3 have similar rates of reactivation (~14.5 h). DMP-777 was evaluated in a series of phase I and phase II clinical trials in cystic fibrosis patients [84]. DMP-777 clearly satisfies the quantum proteolysis hypothesis proposed by Ed Campbell [85]. The ability of DMP-777 to inhibit azurophilic granule NE and PR3 prior to neutrophil activation at a site of inflammation could be an important clinical development property. We propose that inhibitors possessing the properties of DMP-77 have significant advantages over competitive non-cell penetrant inhibitors that do not satisfy the quantum proteolysis hypothesis. Recently, it has become increasingly clear that inhibition of both NE and PR3 with low molecular weight inhibitors will be required to effectively treat AATD [86]. If so, this raises doubts about the potential effectiveness of AZD8668 and BAY85-8501 currently undergoing clinical trials in AATD.

**Figure 3:**
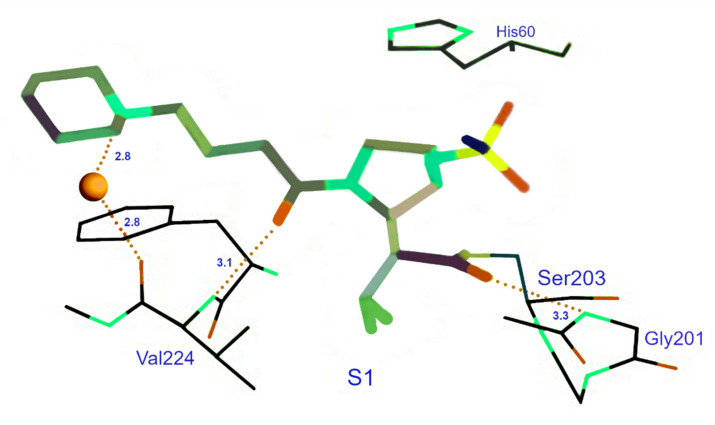
Crystal structure of GW-311616 complexed with porcine pancreatic elastase.

The final inhibitor discussed here is GW-311616, a *trans*-lactam. Research on the interaction between porcine pancreatic elastase and GW-311616 revealed interactions via a covalent bond with Ser203 with NE. Besides the covalent bond, hydrogen bonds are formed between several nitrogen and oxygen atoms (see Figure 3).

Most of these interactions take place in S1, albeit some hydrophobic interactions involving S4 have also been identified. Because porcine pancreatic elastase and NE have very similar structures, NE is likely to have the same interactions with GW-311616. This inhibitor, with a Ki-value of 0.31 nM, is selective for NE over Cath G, trypsin, plasmin, chymotrypsin, and tissue plasminogen activator. Its selectiveness for PR3 is unknown. Just like DMP777, GW-311616 is cell permeable and potently inhibits NE intracellularly. GW-311616 also essentially acts as an irreversible inhibitor, so the duration of the inhibition is independent of the half-life of this inhibitor [87].

In the paragraph above, several types of synthetic NE inhibitors were discussed. In addition to the fact that these inhibitors were developed to target NE activity in patients with excessive activity of this neutrophil-derived enzyme, such inhibitors are also very useful for determining the previously discussed novel assays that detect footprints of NE or PR activity. For this purpose, the most favourable characteristics an inhibitor can have is likely to be cell permeable because in that way most of NE and/or PR3 is inhibited, and reactivity with other proteases will be noticed most easily. Furthermore, inhibitors that can inhibit both NE and PR3 might also prove useful to determine whether other proteases can cleave the neoepitopes generated by NE and PR3 activity. However, when developing an assay specific for either NE or PR3, a single-protease inhibitor might be desirable. The Ki-value should preferably be as low as possible because the Ki is a ratio of the rate constants Koff/Kon. A lower Ki therefore means that the inhibitor is complexed with the protease for a longer period. Taking all this into account, BAY 85-8501, DMP-777, and GW-311616 are suggested to be able to give the best results.

## 6. Conclusions

In brief, AATD is an inflammatory disease of unopposed proteolysis and the only known genetic trigger of COPD. The mutation underlying this disease causes the AAT levels to be under its protective threshold of 11 µM or totally absent. This results in high active levels of NE and PR3, as these are not properly inhibited, and unrestricted activity of these enzymes may contribute to the development of emphysema and other intra- and extrapulmonary diseases. Augmentation therapy has been proposed as a treatment, but its effectiveness is highly controversial. In this regard, studies into biomarkers NE and PR3 activity have been initiated, which reflect in vivo AAT activity. The most promising biomarkers include neoepitopes generated by NE or PR3 activity on elastin and fibrinogen. What is currently missing is a biomarker of NE and PR3 activity which target a lung-specific protein involved in the pathogenesis of emphysema. With this shortcoming, recent research studies developed two elastin and fibrinogen neoepitopes as biomarkers for the development of NE- and PR-specific enzyme-linked immunosorbent assays (ELISAs). These ELISAs are developed with the purpose of measuring the effects of augmentation therapy in patients with AATD more accurately. Hopefully, their implementation will contribute to the discussion on the effectiveness of this therapy and help in determining the efficiency of novel therapies. Because studies into the development of assays to measure these footprints often require validation of their specificity, seven inhibitors were discussed that can prove useful to determine the selectivity of the fibrinogen assays. Due to the different properties they have, three of these inhibitors were suggested to yield the best results for determining the specificity and validity of the assays. These three inhibitors are BAY 85-8501, DMP-777, and GW-311616. However, because data about all of these inhibitors are lacking, this cannot be stated with absolute certainty. Ultimately, this review pointed out different problems regarding the measurement of efficiency of AATD therapies and provided suggestions on how more accurate tests can be developed. If such advances are being made, therapies can be evaluated better. In the past 25 years, it has not been possible to demonstrate in randomised, placebo-controlled trials of intravenous alpha-1-antitrypsin treatment a clinical meaningful important difference on FEV1 decline in AATD patients with pulmonary emphysema [88]. Most healthcare payers in Northwest European countries refuse to reimburse the high cost of treatment of intravenous augmentation therapy for the above patient population. Efficacy, which was shown in several RCTs with PD15 lung density as an outcome parameter, has been reported [88]. However, they demand clinical trial results which show a protective effect on change in FEV1 or gas transfer in patients with pulmonary emphysema. This has not been demonstrated yet. Several causes might be responsible. For example, the dose of intravenous AAT was not adequately based on the biochemical efficacy of inhibition of targeted proteases like NE or PR3. Another cause of failure to show the efficacy of AAT might be the severity of emphysema present at the start of augmentation therapy, i.e., the level of severity is not susceptible to protect against the decline in FEV1 and/or gas transfer. The usual track of drug development is to determine the dose of drug able to adequately inhibit target enzymes like NE and PR3, both hold responsible for emphysema development. This approach provides information about biochemical efficacy by establishing the dose of protease inhibitor at which the plasma level of fibrinopeptide Aα360 and Aα541, specific for NE and PR3 activity on fibrinogen, respectively, has reached values seen in healthy controls. Better and reliably evaluated therapies might then bring some perspective in (re-)considering to show treatment effects on the decline in FEV1 and gas transfer as outcome parameters in phase III randomised placebo-controlled clinical trials.

## Figures and Tables

**Table 1:** NE and/or PR3 specific inhibitors and their properties.

Inhibitor	Specificity	Permeability	NE K_i_ (nM)
ONO-5046	NE and PR3	Non-cell permeable	200 ± 20
ONO-6818	NE	Non-cell permeable	12.16
AZD9668	NE	Unknown	9.4
BAY-678	NE, PR3 unknown	Unknown	15
BAY 85-8501	NE, PR3 unknown	Unknown	0.08
DMP-777	NE and PR3	Cell permeable	Kinac/Ki = 3800.000 M^−1^ s^−1^
GW-311616	NE, PR3 unknown	Cell permeable	0.31

NE; neutrophil elastase; PR3: proteinase 3; K_i_: inhibition constant; K_inac_: inactivation rate constant.

## Data Availability

Not applicable.

## Abbreviations

AATD	alpha1-antitrypsin deficiency
EL-NE	elastin degradation mediated by NE
PR3	proteinase 3
NE	Neutrophil elastase
AAT	alpha1-antitrypsin
GPA	polyangiitis
COPD	chronic obstructive pulmonary disease
RCL	Reactive centre loop
SNPs	single-nucleotide polymorphisms
FEV1	forced expiratory volume in one second
RCT	randomised controlled clinical trial
